# The Impact of eHealth Interventions on the Improvement of Self-Care in Chronic Patients: An Overview of Systematic Reviews

**DOI:** 10.3390/life12081253

**Published:** 2022-08-17

**Authors:** Erika Renzi, Valentina Baccolini, Giuseppe Migliara, Corrado De Vito, Giulia Gasperini, Angelo Cianciulli, Carolina Marzuillo, Paolo Villari, Azzurra Massimi

**Affiliations:** 1 Department of Public Health and Infectious Diseases, Sapienza University of Rome, 00185 Rome, Italy; 2 Department of Translational and Precision Medicine, Umberto I Teaching Hospital, 00161 Rome, Italy; 3 Department of Biomedicine and Prevention, University of Rome Tor Vergata, 00133 Rome, Italy

**Keywords:** eHealth, self-care, chronic diseases

## Abstract

Promoting self-care is one of the most promising strategies for managing chronic conditions. This overview aimed to investigate the effectiveness of eHealth interventions at improving self-care in patients with type-2 diabetes mellitus, cardiovascular disease, and chronic obstructive pulmonary disease when compared to standard care. We carried out a review of systematic reviews on PubMed, Scopus, Cochrane, PsychInfo, and CINAHL. AMSTAR-2 was used for quality appraisal. Eight systematic reviews (six with meta-analysis) were included, involving a total of 41,579 participants. eHealth interventions were categorized into three subgroups: (i) reminders via messaging apps, emails, and apps; (ii) telemonitoring and online operator support; (iii) internet and web-based educational programs. Six systematic reviews showed an improvement in self-care measurements through eHealth interventions, which also led to a better quality of life and clinical outcomes (HbA1C, blood pressure, hospitalization, cholesterol, body weight). This overview provided some implications for practice and research: eHealth is effective in increasing self-care in chronic patients; however, it is required to designate the type of eHealth intervention based on the needed outcome (e.g., implementing telemonitoring to increase self-monitoring of blood pressure). In addition, there is a need to standardize self-care measures through increased use of validated assessment tools.

## 1. Introduction

The sustainability of health systems depends to an extent on the ability of individuals to manage chronic diseases by taking responsibility for and participating in their care process [1,2]. In this context, continuity of care is a crucial element in the management of chronic diseases [3], particularly in the context of primary healthcare [4], where there is a need to promote a change in the care models adopted and to provide dedicated workforces. A transition is required toward a proactive approach that helps patients to achieve a higher level of autonomy in the management of their health conditions and that supports their self-care [5]. This approach can be effective at maintaining and improving health and quality of life and reduces complications, hospital admissions, and mortality [6,7].

eHealth, which is defined by WHO as the “use of information and communication technologies for health” [8], could be a fundamental tool for improving patient-centered care in health systems and self-care of non-communicable diseases (NCDs). Technologies that allow tracking and interventions and can be used by both healthcare professionals and patients/assistants include laptops, smartphones, Fitbit units, tablets, wearable sensors, videoconferencing, and GIS [9,10]. eHealth interventions for NCDs represent an opportunity to facilitate communication, stimulate the demand for services, and increase access to health information for disease management [11,12]. The use of digital technologies in the health context is a priority issue, especially considering the recent COVID-19 pandemic [13], which highlighted the need to implement sustainability of healthcare systems through digitization to enhance continuity of care. Indeed, the COVID-19 pandemic has led to a deterioration in self-care for patients suffering from NCDs [14], following a widespread disruption in chronic disease management services worldwide. In a WHO survey of the ability of different countries to address and respond to the growing burden of NCDs, 122 countries reported that the pandemic had caused delays and/or disruptions in their health services for chronic patients suffering from NCDs in 31% to 65% of cases. Services responsible for the management of patients with diabetes mellitus (T2DM), cardiovascular disease (CVD), and chronic obstructive pulmonary disease (COPD) showed a significant deterioration compared to baseline [15].

The implementation of eHealth and digital health measures appears to offer a viable means of improving the resilience of national health systems [16,17,18]. An understanding of the evidence on the effectiveness of eHealth interventions for the most common NCDs, with a focus on enhancing self-care, is crucial when planning and implementing person-centered care and interventions that involve patients in the management of their disease. However, most published systematic reviews have focused only on specific chronic diseases or single clinical outcomes (e.g., patients with heart failure, blood pressure levels). Here, our overview of systematic reviews aims to investigate the effectiveness of eHealth interventions at improving self-care in patients with chronic conditions, specifically those with type-2 diabetes mellitus, chronic obstructive pulmonary disease, and cardiovascular disease, when compared to standard care.

## 2. Materials and Methods

### 2.1. Selection Criteria and Search Strategy

We carried out a review of systematic reviews using the methodology of the Joanna Briggs Institute [19] to evaluate the efficacy of eHealth interventions in primary care, compared to standard care, at improving self-care in adult patients (>18 years old) with a diagnosis of type-2 diabetes mellitus, cardiovascular disease, or chronic obstructive pulmonary disease.

The primary outcome was the improvement of self-care levels in terms of self-maintenance, self-monitoring, and self-management, based on the definition provided by the middle-range theory of self-care of chronic illness [6] when associated with eHealth interventions that were evaluated through validated measurement tools. Secondary outcomes concerned the association between eHealth interventions and the improvement of observer-related outcomes (OROs) and patient-reported outcomes (PROs) [20].

Due to the recent implementation of technologies in healthcare, we limited our search to the last ten years (2010 to July 2020). We included only three groups of NCDs: T2DM, CVD, and COPD, which are the most common NCDs and are responsible for the majority of global deaths. In addition, due to the characteristics of these diseases (long duration and need for continuity of care), they were most affected during the pandemic by interruption or delay in the delivery of routine health services [15].

The search covered five electronic databases: PubMed, Cumulative Index to Nursing and Allied Health Literature (CINAHL), Scopus, PsycINFO, and the Cochrane Library. A manual search was performed through reference lists and relevant journals (JMIR). The search strategy keywords were based on the middle-range theory of self-care for chronic illness [6] (the search strategy is fully reported in Supplementary File S1).

The main inclusion criteria and their definitions [21,22,23,24,25] are detailed in Table 1. In particular, we included systematic reviews with or without meta-analysis of randomized controlled trials (RCTs), quasi-experimental studies, and cohort studies published in English or Italian and showing studies evaluating self-care using validated measurement tools. We excluded systematic reviews focused only on specific populations (e.g., pregnant women with diabetes, minorities). The reasons for the exclusion of specific populations are related to the difficulties of applying results to the general population with a chronic condition because specific populations are also characterized by peculiar features that distinguish them from other patients with NCDs (e.g., socioeconomic, geographic, and clinical characteristics).

### 2.2. Data Extraction and Quality Assessment

Two reviewers independently screened the records. In case of disagreement that was not solved via consensus, a third reviewer arbitrated the decision process.

Articles were also selected via manual search from the reference list. For data extraction, we used a form that included the following features: population demographics, patient diseases (T2DM, COPD, CVD), eHealth providers, measurement tool, setting, primary outcome, in terms of self-care maintenance, self-care monitoring, and self-care management (Table 1 for definition), secondary outcomes and type of eHealth intervention. The latter, defined as the activities included in “telemedicine” according to the WHO classification of digital health interventions [26], were classified on the basis of the main component of eHealth technologies used to achieve the goal as reported in the included systematic review (e.g., goal: monitor vital signs, eHealth: telemonitoring; goal: improve therapeutic adherence, eHealth: reminders). Generally, eHealth activities include remote monitoring and data transmission, consultancy with remote health workers, and monitoring or training activities through online educational programs. When starting from this classification, three categories were identified by the end of the process: (i) reminders via SMS, MMS, messaging apps, emails, and/or mobile apps; (ii) telemonitoring and online operator support; (iii) internet and web-based educational programs for smartphones, PCs, apps.

Two reviewers independently assessed the methodological quality of the systematic reviews included in our overview using the updated version of A Measurement Tool to Assess Systematic Reviews (AMSTAR-2) [27], a 16-point tool designed for this purpose. Any disagreements were resolved by discussion among reviewers.

## 3. Results

### 3.1. Main Characteristics of the Included Studies

**Figure 1:**
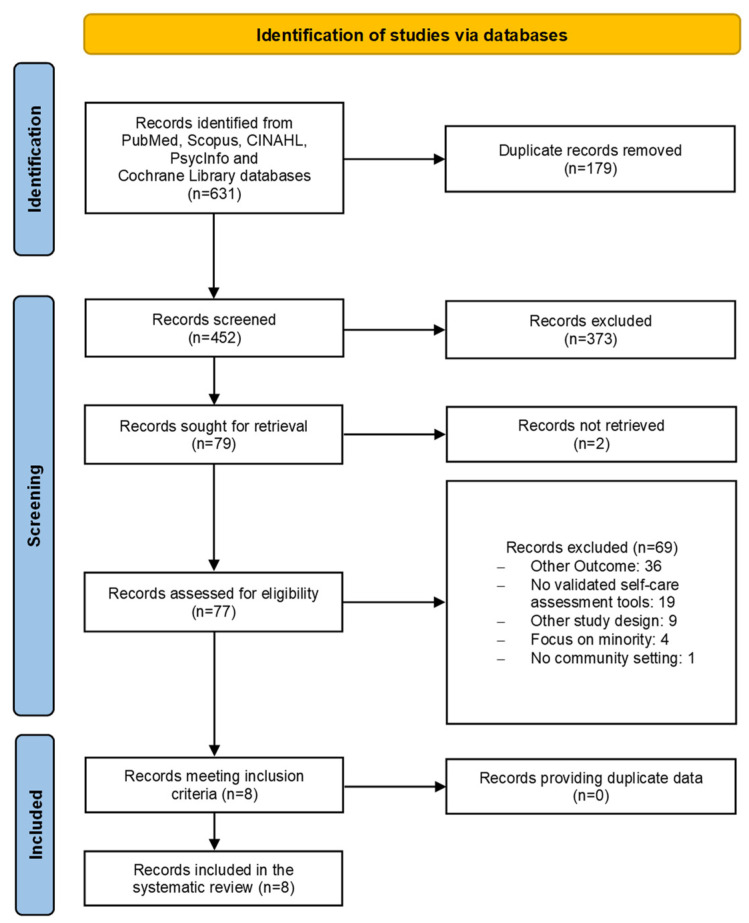
Flowchart diagram of study selection method.

The selection process (title, abstract and full text) and the main reasons for full-text exclusion are shown in the flowchart in Figure 1 and were performed according to PRISMA (Preferred Reporting Items for Systematic Reviews and Meta-Analysis) [28]. Briefly, 637 articles were initially identified, of which, after the removal of duplicates, 452 papers went to the screening phase. Screening by title and abstract yielded 77 articles that were assessed for eligibility. A total of eight articles [29,30,31,32,33,34,35,36], comprising six systematic reviews with meta-analyses [29,30,31,32,33,36] and two systematic reviews [34,35], were finally included.

The reviews encompassed 282 RCTs, one quasi-experimental study, and three cohort studies with a total of 41,579 participants aged 18 to 75 years. Three reviews included patients with CVD [30,32,36], one review concerned patients with T2DM [34], and one review included patients with COPD [33]. The other three systematic reviews were not related to a specific disease but included studies with patients who had at least one of the major chronic diseases (CVD, T2DM, or COPD) [29,31,35].

The eHealth interventions were mostly led by multi-professional teams. Healthcare professionals involved in the delivery of eHealth interventions were largely nurses, physicians, pharmacists, and research staff.

The self-care assessment questionnaires used in the studies covered by the systematic reviews were heterogeneous; the principal questionnaires used were as listed below: COPD-Self-Care Self-Efficacy Scale (SCES), Summary of Diabetes Self-Care Activities Measure (SDSCA), European Heart Failure Self-care Behaviour Scale (EHFScBS) and Self-Care Heart Failure Index (SCHFI).

The results of the AMSTAR-2 quality assessment show that, of the eight systematic reviews included, six were of high quality [29,30,31,32,33,36], and two were of critically low quality [34,35]. The main reason for the “critically low quality” classification of these two systematic reviews was the absence or incomplete implementation of methodological quality assessment of the single studies (item nine of the AMSTAR 2 tool). Detailed results of the quality assessment are reported in Supplementary File S2. A summary of the main characteristics of the included systematic reviews is reported in Table 2.

### 3.2. Types of eHealth Intervention

As already described, the eHealth interventions, even when multicomponent, have been categorized on the basis of the leading technological component characterizing the intervention itself and its main purpose.

1. Reminders via SMS, MMS, messaging apps, emails, and/or mobile apps (abbr. reminders): these interventions consisted of short-message reminders sent by healthcare providers through messaging apps, SMS, MMS, and/or emails with the aim of improving disease awareness and self-care of the chronic illness, and to remind individuals of therapy and daily activities (e.g., physical activity, daily glycemic control). Messaging apps allowed the person to communicate and give real-time feedback to the support operators and also facilitated emergency management by physicians and nurses. Two systematic reviews with meta-analysis [29,30] evaluated the effectiveness of reminder interventions in improving self-care. The eHealth interventions were compared with a traditional care approach that included routine home visits and face-to-face delivery of information only.
2. Telemonitoring and online operator support (abbr. telemonitoring): this method involves the patient transmitting clinical and physiological data via a phone or web-based automated electronic devices to healthcare professionals. Two systematic reviews with meta-analysis [31,32] examined telemonitoring alone or in association with videoconference educational sessions and online real-time operator support for symptom control. A comparison was made with standard care, which included face-to-face care, phone consultation, and routine visits.
3. Internet and web-based educational programs for smartphones, PCs, and apps (abbr. web-based education): these interventions consisted of structured online or offline programs designed to promote self-care using a set of resources that the patient must consult to achieve certain objectives. Four systematic reviews with [33,36] or without meta-analysis [34,35] evaluated this type of eHealth intervention. Standard care included no intervention, face-to-face interventions, education group sessions, and paper-based education materials.

### 3.3. Self-Care Improvements

Improvements in self-care measurements were associated with an eHealth intervention in six of the eight systematic reviews [29,31,32,33,34,35]. Interventions led by multi-professional teams reported more effective results in improving self-care than eHealth interventions led by single professionals and/or research staff [32,34,35]. The overview of the effectiveness of eHealth interventions in improving self-care is described in the following paragraph based on the classification given in Table 1 (self-maintenance, monitoring, and management). Table 3 summarizes the effectiveness of eHealth interventions in improving self-care.

#### 3.3.1. Self-Care Maintenance

Self-care maintenance was investigated in four systematic reviews (two with meta-analysis) [33,34,35,36] and consisted of interventions delivered via web-based education to a total of 15,441 patients. COPD patients registered a significant improvement in terms of self- maintenance, especially in terms of adherence to physical activity and stability of mental health [33]. These results were not confirmed in another systematic review, but this was one of the reviews assigned a “critically low quality” score in the AMSTAR assessment [35]. Web-based education also provided a statistically significant improvement in T2DM patients [34], especially in health education, on topics such as diet and how to monitor blood sugar. Improved self-care maintenance as a result of these eHealth interventions was especially marked in the elderly and those requiring home care [33].

#### 3.3.2. Self-Care Monitoring

Self-care monitoring was evaluated in three systematic reviews [29,31,32] with a total of 23,291 patients. One systematic review included reminder interventions and showed a positive improvement in self-care monitoring in patients with T2DM [29], especially for monitoring blood sugar and weight. For telemonitoring, we included two systematic reviews [31,32], where we recorded an improvement in self-care monitoring only in patients with CVD for the daily assessment of blood pressure values, especially when interventions were provided by a multi-professional team (physician, nurse, pharmacist) [32].

#### 3.3.3. Self-Care Management

Self-care management was evaluated in all the systematic reviews included here. Two systematic reviews with meta-analysis [29,30], with a total of 4071 patients, evaluated self-care management improvements with the use of reminders. No difference was found between the control and intervention groups in patients with COPD and CVD [29,30], whereas a statistically significant improvement was found in patients with T2DM [29] (increased self-management capacity in “Self-Efficacy for Diabetes—SED”—Mean Difference 6.10, 95% CI 0.45 to 11.75), particularly in patients of younger age and those requiring home care [29].

The effect of telemonitoring on self-care management of chronic diseases was studied in two systematic reviews with a total of 23,109 patients [31,32]. Only one systematic review showed significant improvements in self-care management in patients with CVD (heart failure) [32]. No difference was found between standard care and the experimental group for patients with T2DM and COPD [31].

The impact of web-based education on self-management was assessed by four systematic reviews (two with meta-analysis) [33,34,35,36] with a total of 15,441 patients. Only COPD patients recorded positive results for all dedicated programs, with statistically significant improvements in self-management of consulting behaviors, such as speaking to a healthcare provider if coughing/breathlessness increases [33,35].

### 3.4. Secondary Outcomes

Table 4 summarizes the effectiveness of eHealth interventions on secondary outcomes.

#### 3.4.1. Observer-Reported Outcomes

- Blood pressure levels: three systematic reviews with meta-analysis [29,30,31] assessed blood pressure levels in a total of 26,118 patients. Reminder interventions yielded statistically significantly lower systolic and diastolic blood pressure values in the experimental group compared to the control group in patients with hypertension. In particular, eHealth interventions significantly decreased the proportion of patients with inadequate blood pressure control (RR: 0.69, 95% CI: 0.57–0.84) [30]; however, no statistically significant changes were recorded in systolic (Mean Difference 1.10, 95% CI −4.37 to 6.57) and diastolic blood pressure (Mean Difference 1.84, 95% CI −2.14 to 5.82) in patients diagnosed with hypertension [29]. Telemonitoring interventions also showed a reduction in systolic and diastolic blood pressure values (Mean Difference—4.33, 95% CI −5.3 to −3.35: Mean Difference—2.75 95%, CI −3.28 to −2.22) in patients with CVD [31].
- HbA1c: this outcome was evaluated in four systematic reviews [29,31,34,35] in a total of 36,192 patients with T2DM. eHealth interventions, including reminders, showed no significant changes in glycemic values between the intervention and control groups (Mean Difference −0.15, 95% CI −0.77 to 0.47) [29]. In contrast, telemonitoring interventions did provide statistically significant improvements in the experimental group (Mean Difference −0.31, 95% CI −0.37 to −0.24) [31]. Two systematic reviews, rated as “critically low” quality according to AMSTAR−2, which analyzed web-based education, yielded a statistically significant improvement in glycemic control in patients with T2DM with or without other chronic conditions [34,35].
- Total cholesterol, LDL, HDL: three systematic reviews evaluated serum cholesterol levels [30,31,35] in 28,806 patients with chronic conditions. No improvement was reported with reminders in terms of total cholesterol (Mean Difference—0.20, 95% CI −0.49 to 0.08, *p* = 0.16), LDL (Mean Difference −0.14, 95% CI −0.39 to 0.11, *p* = 0.27) and HDL (Mean Difference −0.01, 95% CI −0.11 to 0.10, *p* = 0.92) [30]. However, significantly lower LDL cholesterol values were reported in patients with CVD following supervision via telemonitoring (LDL, Mean Difference 12.45, 95% CI −14.23 to −10.68; *p* < 0.00001) [31]. Another positive effect on LDL values was recorded with interventions using the internet and web-based education, although this review was rated as of “critically low quality” [35].
- Peak oxygen: One systematic review was included with a total of 182 patients, in which the chosen eHealth intervention was the use of reminders. Peak oxygen levels were significantly higher in the intervention group of COPD patients [29].
- Body weight: A single review investigated this outcome in patients with CVD [30]. Reminders were associated with a statistically significant reduction in body mass index (Mean Difference −1.08, 95% CI −2.04 to −0.13).
- Hospitalizations: This outcome was investigated in three of the eight reviews [29,32,36] that included 2165 patients with CVD (heart failure) and T2DM. One review of the use of reminders showed a reduction in emergency hotline use for re-hospitalizations in T2DM patients (RR 0.32, 95% CI 0.09 to 1.08) [29]. One review of telemonitoring interventions showed a statistically significant reduction in heart failure-related hospitalizations (RR 0.85, 95% CI 0.77 to 0.93) [32], but another review showed no such difference (OR 0.74, 95% CI 0.52 to 1.06) [36].
- All-cause mortality: Two systematic reviews with meta-analysis investigated this outcome, both concerning telemonitoring interventions [31,32]. One of the reviews showed positive results in patients with heart failure (RR 0.80, 95% CI 0.68 to 0.94) [32]. However, no statistical significance in all-cause mortality was found in a meta-analysis (RR 0.89, 95% CI 0.76 to 1.03, *p* = 0.12) of patients with COPD, T2DM, and heart failure [31].

#### 3.4.2. Patient-Reported Outcomes

- Quality of life (QoL): Five of the eight systematic reviews evaluated improvement in QoL by means of the SF-36 and SF-12 Health Status Questionnaires and the Kansas City Cardiomyopathy Questionnaire in a total of 27,457 patients [31,32,33,35,36]. Telemonitoring interventions were effective for CVD patients [33], particularly in the case of heart failure [32]. No difference was recorded between experimental and control groups when web-based educational programs were used [35,36].
- Adherence to medication regime: The effectiveness of reminders and web-based education interventions at ensuring adherence to a prescribed medication regime was evaluated by two systematic reviews [29,35]. No significant changes in chronic patients with T2DM, COPD, and CVD were uncovered.

## 4. Discussion

Healthcare systems worldwide face new health and organizational challenges as a result of two distinct phenomena: an aging population with an increased prevalence of chronic diseases and the need for healthcare systems to migrate outside of hospitals to promote proactive medicine and community support [37,38,39]. Chronic patients are, in fact, challenged with both an increase in their overall health needs and the necessity to guarantee continuity of care [40,41]. Primary care settings can help achieve these objectives by granting patients access to healthcare services and facilitating continuity of care [42].

According to the results of our overview of systematic reviews, community-wide eHealth interventions can indeed have a positive impact on self-care in patients with chronic diseases [29,31,32,33,34,35]. The eHealth approach also allows a higher degree of continuity of care than traditional methods delivered in community settings and/or at home and makes it possible to provide interventions founded on personalized care [31,32,35,43]. This is especially true in light of the recent COVID-19 pandemic [16], which highlighted, even more, the need to maintain close contact with chronic patients [44,45] to offer as much continuity of care as possible, despite a widespread reduction in the availability of access to healthcare services [16]. In fact, the eHealth interventions included in this overview appeared to be effective at improving self-care in chronic patients in six of the eight systematic reviews retrieved [29,31,32,33,34,35]. Self-care interventions in chronic patients were found to be effective when consistently monitored and maintained with the support of health services [46].

Thus, eHealth helps chronic patients in self-care by:

- Improving behavior that maintains physical and emotional stability (self-maintenance). This is particularly effective in COPD patients who use web-based education to ensure continuity in educational programs that maintain their physical and emotional status and allow them to control respiratory exacerbations [33,47,48];
- Providing early recognition of those signs and symptoms that suggest a deterioration in the patient’s own health status (self-monitoring). In fact, this review has shown that the use of telemonitoring with the support of the operator, or reminders in patients with T2DM and CVD, allows early recognition of a deterioration in health status and prevents acute episodes, especially in patients with the decompensated disease [29,32,49,50];
- Allowing prompt action by means of lifestyle changes (self-management) [30,31,32,34,51] in all patients, particularly when using web-based education programs.

Our overview also showed that eHealth effectively enhances OROs and PROs in chronic patients. For example, telemonitoring interventions improve the quality of life for all chronic diseases and reduce hospitalization and mortality in patients with CVD [29,31,32,33,52,53]. Another interesting observation that emerged from this overview was that, although reminders are widely used to improve adherence to their medication regime in patients with chronic diseases, this improvement declined in the long term. Thus, patients using eHealth interventions for more than six months tended to return to “bad habits” once the novelty of telemedicine had worn off [29,35,54]. In fact, the duration of the intervention and engagement with it are also important factors that influence its effectiveness [55,56]. This evidence, in line with the literature, highlights the importance of the role of healthcare workers in encouraging patient adherence to eHealth programs [57,58].

Regarding the role of healthcare workers, this summary emphasizes the fact that eHealth interventions are most effective at improving self-care when they are led by multidisciplinary teams, especially when such teams work in primary care [30,32,34,35,36,59,60,61,62]. This is probably because specialized multidisciplinary teams can address both health and social health issues, ensuring that care is personalized and based on the perceived needs of the patient [63].

In conclusion, this overview carries some implications for practice, proving that eHealth is effective in increasing self-care in chronic patients with T2DM, CVD, and COPD; however, one must first be able to designate the most appropriate type of eHealth intervention based on the outcome to be achieved (e.g., implementing telemonitoring to increase self-monitoring of blood pressure). The results of this synthesis could help health care providers choose the most effective, outcomes-based eHealth interventions. In addition, this overview that included most of the major chronic diseases provided an overview of the effectiveness of eHealth on improving self-care, considering two aspects: (i) most population with chronic disease lives with multimorbidity, and designing an eHealth intervention on the basis of pathology could be a limitation (ii) eHealth interventions in increasing self-care should not be limited to disease, as self-care is a fundamental ability of patients with NCDs to live with their new life condition.

Finally, this overview of evidence brings to light two implications for the research: first, we observed that few systematic reviews in the literature use validated tools to assess the effectiveness of eHealth interventions in improving self-care. This might be because many of the self-care tools currently available have been developed for specific diseases and thus have limited applicability to other conditions, and also because transferring the data to the appropriate electronic platform can be a complex process [64]. Unfortunately, these limitations make it impossible to systematically evaluate those results that are not supported by standardized, validated instruments. Therefore, the quality of evidence would be markedly improved by the use of such standardized instruments across the scientific community to systematically evaluate self-care in all populations.

Secondly, none of the systematic reviews in our survey assessed the eHealth literacy of the patients involved, despite its importance for effective use of the interventions. The literature shows that people with high levels of eHealth literacy are empowered and enabled to fully participate in health decisions informed by eHealth resources and technologies [65]. Where eHealth literacy is at a low level, e.g., in elderly or rural populations [66], the ability to participate in eHealth interventions that aim to improve self-care is known to be reduced. If eHealth literacy levels are not assessed or if only technologically competent participants are selected, the results of any eHealth study or program are likely to be affected.

Finally, it should be noted as a limitation that this systematic review included only articles published in the last decade and up to July 2020. However, this choice allowed us to synthesize the most recent evidence by including systematic reviews with RCTs in chronic patient populations that were not affected by organizational changes resulting from the COVID-19 pandemic and are therefore more representative of care delivered in non-emergency settings.

## 5. Conclusions

eHealth interventions represent a means by which self-care and disease management in chronic patients can be increased. These interventions could also be applicable to the problems encountered during the COVID-19 pandemic. They might allow for much greater continuity of care during an emergency and non-emergency situation, supporting the sustainability of health care systems by reducing avoidable hospitalizations and re-hospitalizations and managing patients in primary care. However, it will be necessary to implement studies that investigate the effect of health inequality on the use of such eHealth interventions, considering the cost and availability of the electronic tools that this type of care requires. Furthermore, systematic reviews of higher methodological quality and with larger patient populations, particularly COPD patients, are urgently needed to assess the efficacy of eHealth in self-care programs. Wider adoption of standardized, validated tools for self-care assessment is also needed to achieve greater homogeneity of self-care measures, given that current evidence is based on a limited number of large studies of mixed methodological quality that can lack reliable self-care assessment tools.

## Figures and Tables

**Table 1:** Main inclusion criteria and their definitions.

Inclusion Criteria	Definition	
**Population**	*People with* *T2DM,* *CVD,* *COPD*	The following are WHO definitions of the NCDs covered in this overview: (i) T2DM is a chronic, metabolic disease characterized by elevated levels of blood glucose (or blood sugar), which leads over time to serious damage to the heart, blood vessels, eyes, kidneys and nerves [21]. (ii) CVD are a group of disorders of the heart and blood vessels and include coronary heart disease, cerebrovascular disease, rheumatic heart disease and other conditions [22]. (iii) COPD is a disease characterized by chronic airflow limitation and a range of pathological changes in the lung [23].
**Intervention**	*eHealth interventions*	eHealth is an emerging field at the intersection of medical informatics, public health and business; it refers to health services and information delivered or enhanced through the internet and related technologies [24].
**Comparison**	*Standard care*	All interventions carried out without the use of the above digital technologies, particularly involving controlled visits (hospitals, outpatient clinics, general practitioners), paper-based information, and face-to-face interventions.
**Outcome**	*Self-care*	A process of maintaining health through health-promoting practices and managing illness. The middle range theory defines three key concepts: Self-care maintenance is defined as those behaviors used to maintain physical and emotional stability (daily physical activity). Self-care monitoring refers to the process of observing oneself for changes in signs and symptoms (for example, being able to monitor vital signs). Self-care management is defined as the response to signs and symptoms when they occur (for example, insulin administration in case of hyperglycemia) [6,25].
**Setting**	*Community*	Community setting includes patients’ home, outpatient clinics and pharmacies, primary care clinics and community hospitals.
**Type of study**	*Systematic Review*	We included systematic reviews with or without meta-analysis of RCTs, quasi-experimental studies and cohort studies.

**Table 2:** Main characteristics of the systematic reviews.

Author Year	Studies Included	Participants N (Mean Age)	NCDs	Intervention	Control	Assessment Tools	eHealth Providers	Setting	Primary Outcome	AMSTAR
**De Jongh,** **2012** [29]	Systematic review and meta-analysis of 4 RCTs	182 (44.7)	T2DM, CVD, COPD	Reminders	Standard care	(i) Summary of Diabetes Self-Care Activities Measure (SDSCA) (ii) Self-Efficacy for Diabetes test	(i) Research staff nurse and physician (2 RCTs) N.A. (2 RCTs)	Home	1. Health outcomes. 2. Capacity to self-manage long-term conditions	High Quality
**Ma, 2019** **[30]**	Systematic review (15 RCTs) and meta-analysis of 14 RCTs	3889 (58)	Hypertension	Reminders	Standard care Attention control	Hill-Bone Compliance to High Blood Pressure Therapy Scale	(i) Nurse (5 RCTs). (ii) Physician (4 RCTs) (iii) Pharmacist (3 RCTs) (iv) Multi-professional team (3 RCTs)	(i) Primary care clinics (ii) Community health centers (iii) Clinics	1. Delivery mode and strategies of current eHealth interventions 2. Effectiveness of eHealth interventions on blood pressure control, self-care, and behavioral outcomes 3. Psychosocial well-being	High Quality
**Flodgren,** **2015 [31]**	Systematic review of 93 RCTs and meta-analysis (66 RCTs on self-care)	22,047 (N.A.)	T2DM, COPD, Heart failure (27 studies on other chronic diseases)	Telemonitoring	Standard care Face-to-face Phone consultation	(i) European Heart Failure Self-care Behaviour Scale (EHFScBS) (ii) Self-Care Heart Failure Index (SCHFI)	N.A.	(i) Primary care clinics (ii) Community health centers (iii) Clinics	1. Mortality. 2. Disease- specific and general measures of health status 3.Healthcare resource use 4. Costs	High Quality
**Inglis, 2015** [32]	Systematic review of 41 RCTs and meta- analysis (7 RCTs on self-care)	1062 (57.78)	Heart failure	Telemonitoring	Standard care	(i) Self-Care Heart Failure Index (SCHFI) (ii) European Heart Failure Self-care Behaviour Scale (EHFScBS)	(i) Multi-professional team (41 RCTs)	Home setting	1. All-cause mortality 2. All-cause hospitalizations 3. Heart failure- related hospitalizations	High Quality
**McCabe, 2017** [33]	Systematic review of 3 RCTs and meta-analysis	557 (64)	COPD	Web-based education	Standard care Face-to-face and/or hard copy/digital documentary educational/ self-management support	(i) St. George’s Respiratory Questionnaire (SGRQ) (ii) COPD-Self-Care Self-Efficacy Scale (SCES)	(i) Research staff (2 RCTs). (ii) Activity coach, researchers (1 RCT)	(i) Home setting (ii) Primary care clinics (iii) Community health centers (iv) Clinics	1. Hospital admissions 2. Acute exacerbations 3. Health-related quality of life	High Quality
**Chrvala, 2016** [34]	Systematic review of 120 RCTs	11,093 (65.18)	T2DM	Web-based education	Standard care Waiting list	Summary of Diabetes Self-Care Activities Measure (SDSCA)	(i) Multi-professional team (53 RCTs) (ii) Physician (13 RCTs) (iii) Nurse (2 RCTs) (iv) Pharmacist (3 RCTs) (v) Nurses and physician (49 RCTs)	(i) Primary care clinics (ii) Community health centers. (iii) Clinics	Diabetes self- management and effect on glycemic control	Critically-Low Quality
**Rush, 2018** [35]	Systematic review of 16 studies (12 RCTs, 3 cohort studies, 1 quasi-experimental study)	2870 (54)	T2DM, COPD, Heart failure	Web-based education	Standard care Routine visits. face-to-face education Paper copies of materials	(i) Summary of Diabetes Self- Care Activities Measure (SDSCA) (ii) Chronic Respiratory Questionnaire dyspnea (CRQ-D) subscale (iii) European Heart Failure Self-care Behaviour Scale (EHFScBS)	(i) Nurse (4 RCTs) (ii) Physician (3 RCTs) (iii) Research staff (1 RCT) (iv) Multi-professional team (8 RCTs, quasi- experimental, observational, cohort study)	(i) Home setting (ii) Primary care clinics	The efficacy of telehealth-delivered Educational approaches for patients with chronic diseases	Critically-Low Quality
**Allida, 2020** [36]	Systematic review and meta-analysis of 5 RCTs	921 (67.5)	Heart failure	Web-based education	Standard care	(i) European Heart Failure Self-care Behaviour Scale (EHFScBS) (ii) Self-Care Heart Failure Index (SCHFI)	(i) Multi-professional team (3 RCTs) (ii) Research nurse (1 RCT) N.A. (1 RCT)	Home settings	1. Heart-failure knowledge 2. Self-efficacy 3. Self-care 4. Adverse events	High Quality

**Table 3:** Summary of the effectiveness of eHealth interventions at self-care improvement.

Primary Outcome	Type of Intervention	Reference	Chronic Disease	Result	AMSTAR 2
**Self-care improvement**					
*Self-maintenance*	Web-based education	McCabe, 2017 [33]	COPD	+	High Quality
	Web-based education	Chrvala, 2016 [34] *	T2DM	+	Critically Low
	Web-based education	Rush, 2018 [35] *	T2DM-COPD	ns	Critically Low
	Web-based education	Allida, 2020 [36]	CVD	ns	High Quality
*Self-monitoring*	Reminders	De Jongh, 2012 [29]	T2DM	+	High Quality
	Telemonitoring	Flodgren, 2016 [31]	T2DM-COPD	ns	High Quality
	Telemonitoring	Inglis, 2015 [32]	CVD	+	High Quality
*Self-management*	Reminders	De Jongh, 2012 [29]	T2DM	+	High Quality
	Reminders	De Jongh, 2012 [29]	COPD	ns	High Quality
	Reminders	Ma, 2019 [30]	CVD	ns	High Quality
	Telemonitoring	Flodgren, 2016 [31]	T2DM-COPD	ns	High Quality
	Telemonitoring	Inglis, 2015 [32]	CVD	+	High Quality
	Web-based education	McCabe, 2017 [33]	COPD	+	High Quality
	Web-based education	Chrvala, 2016 [34] *	T2DM	+	Critically Low
	Web-based education	Rush, 2018 [35] *	COPD	+	Critically Low
	Web-based education	Rush, 2018 [35] *	T2DM	ns	Critically Low
	Web-based education	Allida, 2020 [36]	CVD	ns	High Quality

* No meta-analysis. +: Statistically significant results in favor of the intervention. ns: results not statistically significant. Reminders: Reminders via SMS, MMS, messaging apps, email, and/or mobile apps. Telemonitoring: Telemonitoring and online operator support. Web-based education: Internet and web-based educational programs for smartphones, PCs, and apps.

**Table 4:** Summary of the effectiveness of eHealth interventions at achievement of secondary outcomes.

Outcome Category	Type of Intervention	Reference	Chronic Disease	Result	AMSTAR 2
**Observer-Reported Outcomes**					
*Systolic blood pressure*	Reminders	De Jongh, 2012 [29]	CVD	ns	High Quality
	Reminders	Ma, 2019 [30]	CVD	+	High Quality
	Telemonitoring	Flodgren, 2015 [31]	CVD	+	High Quality
*Diastolic blood pressure*	Reminders	De Jongh, 2012 [29]	CVD	ns	High Quality
	Reminders	Ma, 2019 [30]	CVD	+	High Quality
	Telemonitoring	Flodgren, 2015 [31]	CVD	+	High Quality
*HbA1c*	Reminders	De Jongh, 2012 [29]	T2DM	ns	High Quality
	Telemonitoring	Flodgren, 2015 [31]	T2DM	+	High Quality
	Web-based education	Chrvala, 2016 [34] *	T2DM	+	Critically Low
	Web-based education	Rush, 2018 [35] *	T2DM	+	Critically Low
*Total cholesterol*	Reminders	Ma, 2019 [30]	CVD	ns	High Quality
	Web-based education	Rush, 2018 [35] *	CVD, T2DM	+	Critically Low
*LDL cholesterol*	Reminders	Ma, 2019 [30]	CVD	ns	High Quality
	Telemonitoring	Flodgren, 2015 [31]	CVD	+	High Quality
	Web-based education	Rush, 2018 [35] *	CVD, T2DM	+	Critically Low
*HDL cholesterol*	Reminders	Ma, 2019 [30]	CVD	ns	High Quality
*Peak oxygen*	Reminders	De Jongh, 2012 [29]	COPD	+	High Quality
*Body weight*	Reminders	Ma, 2019 [30]	CVD	+	High Quality
*Hospitalizations*	Reminders	De Jongh, 2012 [29]	CVD, T2DM, COPD	+	High Quality
	Telemonitoring	Inglis, 2015 [32]	CVD	+	High Quality
	Web-based education	Allida, 2020 [35]	CVD	ns	High Quality
*All-cause mortality*	Telemonitoring	Flodgren, 2016 [31]	CVD, T2DM, COPD	ns	High Quality
	Telemonitoring	Inglis, 2015 [32]	CVD	+	High Quality
**Patient-Reported Outcomes**					
*Quality of life*	Telemonitoring	Flodgren, 2016 [31]	CVD, T2DM, COPD	+	High Quality
	Telemonitoring	Inglis, 2015 [32]	CVD	+	High Quality
	Web-based education	McCabe, 2017 [33]	COPD	+	Critically Low
	Web-based education	Rush, 2018 [35] *	T2DM, COPD	ns	Critically Low
	Web-based education	Allida, 2020 [36]	CVD	ns	High Quality
*Medication adherence*	Reminders	De Jongh, 2012 [29]	CVD, T2DM	ns	High Quality
	Web-based education	Rush, 2018 [35] *	T2DM, COPD	ns	Critically Low

* No meta-analysis. +: Statistically significant results in favor of the intervention. ns: result not statistically significant. Reminders: Reminders via SMS, MMS, messaging apps, email and/or mobile apps. Telemonitoring: Telemonitoring and online operator support. Web-based education: Internet and web-based educational programs for smartphones, PCs, apps.

## Data Availability

The data presented in this study are available on request from the corresponding author.

## Supplementary Materials

The following supporting information can be downloaded at: https://www.mdpi.com/article/10.3390/life12081253/s1, Supplementary File S1_search strategy; Supplementary File S2_AMSTAR2.0_Checklist.
