# Supplementary material for: The Impact of eHealth Interventions on the Improvement of Self-Care in Chronic Patients: An Overview of Systematic Reviews
Source: Life (Basel). 2022 Aug 17;12(8):1253. doi: 10.3390/life12081253 (PMC9409893; doi:10.3390/life12081253)
Supplement: Supplementary file 1 [file life-12-01253-s001.zip › Supplementary file S1_search strategy.pdf]

Supplementary File S1 – search strategy

Pubmed

(((((((((((((((((((((((("Chronic Disease"[Title/Abstract] OR "chronic disease*"[Title/Abstract]) OR "Diabetes mellitus type 2"[Title/Abstract]) OR "Diabetes mellitus type II"[Title/Abstract]) OR "Diabetes mellitus type two"[Title/Abstract]) OR "type II Diabetes"[Title/Abstract]) OR "type two Diabetes"[Title/Abstract]) OR "Type 2 Diabetes"[Title/Abstract]) OR "Diabetes type 2"[Title/Abstract]) OR "Diabetes type II"[Title/Abstract]) OR "Diabetes type two"[Title/Abstract]) OR "T2D"[Title/Abstract]) OR "T2DM"[Title/Abstract]) OR "COPD"[Title/Abstract]) OR "Chronic obstructive pulmonary disease"[Title/Abstract]) OR "Cardiovascular disease"[Title/Abstract]) OR "cardiovascular disease*"[Title/Abstract]) OR "CVD"[Title/Abstract]) OR "CVD"[Title/Abstract]) OR "Cardiovascular Diseases"[MeSH Terms]) OR "Chronic Disease"[MeSH Terms]) OR "diabetes mellitus, type 2"[MeSH Terms]) AND ((((((((((((((((((((((((((("ehealth"[Title/Abstract] OR "e-health"[Title/Abstract]) OR "Telemedicine"[Title/Abstract]) OR "telemed*"[Title/Abstract]) OR "Telemedicine"[MeSH Terms]) OR "telehealth"[Title/Abstract]) OR "mhealth"[Title/Abstract]) OR "m-health"[Title/Abstract]) OR "mobile health"[Title/Abstract]) OR "digital health"[Title/Abstract]) OR "videoconferenc*"[Title/Abstract]) OR "video conferenc*"[Title/Abstract]) OR "telemonitor*"[Title/Abstract]) OR "tele monitor*"[Title/Abstract]) OR "e-mail"[Title/Abstract]) OR "electronic mail"[Title/Abstract]) OR "short message"[Title/Abstract]) OR "short messaging"[Title/Abstract]) OR "electronic messaging"[Title/Abstract]) OR "SMS"[Title/Abstract]) OR "app"[Title/Abstract]) OR "mobile application"[Title/Abstract]) OR "mobile applications"[Title/Abstract]) OR "phone"[Title/Abstract]) OR "social network"[Title/Abstract]) OR "e-learning"[Title/Abstract]) OR "web-based"[Title/Abstract]) OR "web-based"[Title/Abstract])) AND (("systematic review"[Title/Abstract] OR "systematic review*"[Title/Abstract]) OR "meta analys*"[Title/Abstract])) AND (((("self care"[Title/Abstract] OR "self-monitoring"[Title/Abstract]) OR "self-management"[Title/Abstract]) OR "self-maintenance"[Title/Abstract]) OR "self care"[MeSH Terms])

CINAHL - Cumulative Index to Nursing and Allied Health Literature.
AB "Chronic Disease" OR "chronic disease*" OR "Diabetes mellitus type 2" OR "Diabetes mellitus type II" OR "Diabetes mellitus type two" OR "type II Diabetes" OR "type two Diabetes" OR Type 2 Diabetes" OR "Diabetes type 2" OR "Diabetes type II" OR "Diabetes type two" OR "T2D" OR "T2DM" OR "COPD" OR "Chronic obstructive pulmonary disease" OR "Cardiovascular disease" OR "cardiovascular disease*" OR CVD AND AB "ehealth" OR "e-health"OR "Telemedicine" OR "telemed*" OR "Telemedicine"OR "telehealth"[Title/Abstract]) OR "mhealth" OR "m-health" OR "mobile health" OR "digital health"OR "videoconferenc*" OR "video conferenc*" OR "telemonitor*" OR "tele monitor*" OR "e-mail" OR "electronic mail" OR "short message" OR "short messaging" OR "electronic messaging" OR "SMS" OR "app" OR "mobile application" OR "mobile applications" OR "phone" OR "social network" OR "e-learning" OR "web-based" OR "web-based" AND AB "self care"OR "self-monitoring" OR "self-management" OR "self-maintenance" AND AB "systematic review" OR "systematic review*" OR "meta analys*"

APA PsycInfo
AB "Chronic Disease" OR "chronic disease*" OR "Diabetes mellitus type 2" OR "Diabetes mellitus type II" OR "Diabetes mellitus type two" OR "type II Diabetes" OR "type two Diabetes" OR Type 2 Diabetes" OR "Diabetes type 2" OR "Diabetes type II" OR "Diabetes type two" OR "T2D" OR "T2DM" OR "COPD" OR "Chronic obstructive pulmonary disease" OR "Cardiovascular disease" OR "cardiovascular disease*" OR CVD AND AB "ehealth" OR "e-health"OR "Telemedicine" OR "telemed*" OR "Telemedicine"OR "telehealth"[Title/Abstract]) OR "mhealth" OR "m-health" OR "mobile health" OR "digital health"OR "videoconferenc*" OR "video conferenc*" OR "telemonitor*" OR "tele monitor*" OR "e-mail" OR "electronic mail" OR "short message" OR "short messaging" OR "electronic messaging" OR "SMS" OR "app" OR "mobile application" OR "mobile applications" OR "phone" OR "social network" OR "e-learning" OR "web-based" OR "web-based" AND AB "self care"OR "self-monitoring" OR "self-management" OR "self-maintenance" AND AB "systematic review" OR "systematic review*" OR "meta analys*"

Scopus

(((TITLE-ABS-KEY ("chronic disease" OR "Diabetes mellitus type 2" OR "Diabetes mellitus type II") OR "Diabetes mellitus type two" OR "type II Diabetes" OR "type two Diabetes" OR "Type 2 Diabetes" OR "Diabetes type 2" OR "Diabetes type II" OR "Diabetes type two" OR "T2D" OR "T2DM" OR "COPD" OR "Chronic obstructive pulmonary disease" OR "Cardiovascular disease" OR "cardiovascular disease*" OR "CVD") AND (TITLE-ABS-KEY ("ehealth" OR "e-health" OR "Telemedicine" OR "telemed*" OR "Telemedicine" OR "telehealth" OR "mhealth" OR "m-health" OR "mobile health" OR "digital health" OR "videoconferenc*" OR "video conferenc*" OR "telemonitor*" OR "tele monitor*" OR "e-mail" OR "electronic mail" OR "short message" OR "short messaging" OR "electronic messaging" OR "SMS" OR "app" OR "mobile application" OR "mobile applications" OR "phone" OR "social network" OR "e-learning" OR "web-based" OR "web-based"))) AND (TITLE-ABS-KEY ("self care" OR "self-monitoring" OR "self-management" OR "self-maintenance"))) AND (TITLE-ABS-KEY ("systematic review" OR "systematic review*" OR "meta analys*"))

COCHRANE LIBRARY

AB "Chronic Disease" OR "chronic disease*" OR "Diabetes mellitus type 2" OR "Diabetes mellitus type II" OR "Diabetes mellitus type two" OR "type II Diabetes" OR "type two Diabetes" OR Type 2 Diabetes" OR "Diabetes type 2" OR "Diabetes type II" OR "Diabetes type two" OR "T2D" OR "T2DM" OR "COPD" OR "Chronic obstructive pulmonary disease" OR "Cardiovascular disease" OR "cardiovascular disease*" OR CVD AND AB "ehealth" OR "e-health"OR "Telemedicine" OR "telemed*" OR "Telemedicine"OR "telehealth"[Title/Abstract]) OR "mhealth" OR "m-health" OR "mobile health" OR "digital health"OR "videoconferenc*" OR "video conferenc*" OR "telemonitor*" OR "tele monitor*" OR "e-mail" OR "electronic mail" OR "short message" OR "short messaging" OR "electronic messaging" OR "SMS" OR "app" OR "mobile application" OR "mobile applications" OR "phone" OR "social network" OR "e-learning" OR "web-based" OR "web-based" AND AB "self care"OR "self-monitoring" OR "self-management" OR "self-maintenance" AND AB "systematic review" OR "systematic review*" OR "meta analys*"
