# Supplementary material for: The Impact of eHealth Interventions on the Improvement of Self-Care in Chronic Patients: An Overview of Systematic Reviews
Source: Life (Basel). 2022 Aug 17;12(8):1253. doi: 10.3390/life12081253 (PMC9409893; doi:10.3390/life12081253)
Supplement: Supplementary file 1 [file life-12-01253-s001.zip › Supplementary file S2_AMSTAR2.0_Checklist.pdf]

| AMSTAR 2 - Assessing the Methodological Quality of Systematic Reviews | Chrvala, 2016 | Rush, 2018 | De Jongh, 2012 | Inglis, 2015 | Flodgren, 2016 | McCabe, 2017 | Ma, 2018 | Allida, 2020 |
|---|---|---|---|---|---|---|---|---|
| **Overall** | **Critically Low-quality review** | **Critically Low-quality review** | **High quality review** | **High quality review** | **High quality review** | **High quality review** | **High quality review** | **High quality review** |
| Did the research questions and inclusion criteria for the review include the components of PICO? | Y | Y | Y | Y | Y | Y | Y | Y |
| Did the report of the review contain an explicit statement that the review methods were established prior to the conduct of the review and did the report justify any significant deviations from the protocol? | N | N | Y | Y | Y | Y | Y | Y |

Supplementary File S2: **AMSTAR 2 - Assessing the Methodological Quality of Systematic Reviews**

| | | | | | | | | |
|---|---|---|---|---|---|---|---|---|
| Did the review authors explain their selection of the study designs for inclusion in the review? | N | N | Y | Y | Y | Y | Y | Y |
| Did the review authors use a comprehensive literature search strategy? | PY | PY | Y | Y | Y | Y | Y | Y |
| Did the review authors perform study selection in duplicate? | N | Y | Y | Y | Y | Y | Y | Y |
| Did the review authors perform data extraction in duplicate? | N | Y | Y | Y | Y | Y | Y | Y |

Supplementary File S2: **AMSTAR 2 - Assessing the Methodological Quality of Systematic Reviews**

| | | | | | | | | |
|---|---|---|---|---|---|---|---|---|
| Did the review authors provide a list of excluded studies and justify the exclusions? | N | N | Y | Y | Y | Y | Y | Y |
| Did the review authors describe the included studies in adequate detail? | Y | Y | Y | Y | Y | Y | Y | Y |
| Did the review authors use a satisfactory technique for assessing the risk of bias (RoB) in individual studies that were included in the review? | N | N | Y | Y | Y | Y | Y | Y |
| Did the review authors report on the sources of funding for the studies included in the review? | N | N | Y | Y | Y | Y | Y | Y |
| If meta-analysis was performed did the review authors use appropriate methods for statistical combination of results? | NA | NA | Y | Y | Y | Y | Y | Y |

Supplementary File S2: **AMSTAR 2 - Assessing the Methodological Quality of Systematic Reviews**

| | | | | | | | | |
|---|---|---|---|---|---|---|---|---|
| If meta-analysis was performed, did the review authors assess the potential impact of RoB in individual studies on the results of the meta-analysis or other evidence synthesis? | NA | NA | Y | Y | Y | Y | Y | Y |
| Did the review authors account for RoB in individual studies when interpreting/ discussing the results of the review? | N | N | Y | Y | Y | Y | Y | Y |
| Did the review authors provide a satisfactory explanation for, and discussion of, any heterogeneity observed in the results of the review? | N | N | Y | Y | Y | Y | Y | Y |

| | | | | | | | | |
|---|---|---|---|---|---|---|---|---|
| If they performed quantitative synthesis did the review authors carry out an adequate investigation of publication bias (small study bias) and discuss its likely impact on the results of the review? | NA | NA | Y | Y | Y | Y | Y | Y |
| Did the review authors report any potential sources of conflict of interest, including any funding they received for conducting the review? | N | Y | Y | Y | Y | Y | N | Y |

| |
|---|
| Y = Yes |
| N = No |
| PY = Partial Yes |
| NA = Not available |

Supplementary File S2: **AMSTAR 2 - Assessing the Methodological Quality of Systematic Reviews**
